# Forebrain Ischemia Triggers GABAergic System Degeneration in Substantia Nigra at Chronic Stages in Rats

**DOI:** 10.1155/2010/506952

**Published:** 2010-10-14

**Authors:** B. Lin, S. Levy, A. P. Raval, M. A. Perez-Pinzon, R. A. DeFazio

**Affiliations:** Department of Neurology (D4-5), Cerebral Vascular Disease Research Center, University of Miami Miller School of Medicine, P.O. Box 016960, Miami, FL 33101, USA

## Abstract

The long-term consequences of forebrain ischemia include delayed Parkinson's syndrome. This study revealed delayed neurodegeneration in the substantia nigra 8 weeks after 12.5 minutes of global ischemia in rat brain. Following neuronal loss of 30–40% in central and dorsolateral striatum at day 3, neuronal damage in the substantia nigra (SN) was assessed at 4–8 weeks using immunohistochemistry for glutamate decarboxylase 67 (GAD67), vesicular GABA transporter (VGAT), and calretinin (CR). At day 56, the optical density of GAD67-, but not VGAT-, immunoreactivity in substantia nigra pars reticulata (SNR)—significantly decreased. CR-neurons concentrated in substantia nigra pars compacta (SNC) were reduced by 27% from day 3 (*n* = 5) to day 56 (*n* = 7, ANOVA, *p* < .01). Movement coordination was impaired at day 56, as evaluated using beam-walking test (time-to-traverse 5.6 ± 1.2 sec versus 11.8 ± 5.4 sec; sham versus ischemia, *p* < .05, *n* = 5, and 7, resp.). Our results demonstrate delayed impairment of the GABAergic system components in SN and associated with movement deficits after global ischemia.

## 1. Introduction

In the weeks to months following resuscitation after cardiac arrest, delayed progressive secondary injury (postischemic encephalopathy) gives rise to symptoms associated with Parkinson's syndrome (or called *parkinsonism*). Although the symptoms resemble those of Parkinson's disease (PD), there is no profound loss of dopaminergic neurons in the postischemic brains. The delayed brain injury is the major concern in clinic, because it is much more severe than the early (3–7 days) primary damage after global cerebral ischemia [1–3]. About 30%–80% of survivors of resuscitation suffer progressive brain deterioration accompanied by parenchymal lesions in the weeks following the initial recovery from the acute ischemia [1, 4]. The encephalopathy also happens after heart surgeries [5, 6] and acute hypoxia [7, 8]. The patients may recover without symptoms within 1-2 days after the insult but, in some cases, may then undergo sudden deterioration, including coordination disability, Parkinson's syndrome, severe dementia, and/or spastic paralysis associated with new brain lesions following a symptom-free period averaging 2–4 weeks after forebrain ischemia [2, 6] or hypoxia owing to acute carbon monoxide intoxication [7, 8]. Recent studies revealed that the delayed encephalopathy is a consequence of secondary ischemia [9]. In a rat model of global ischemia, a delayed loss of *γ*-aminobutyric acid (GABA) containing interneurons in the hippocampus coexisted with a decreased seizure threshold [10]. The expression of Parkinsonism in humans and the delayed vulnerability of the GABAergic interneurons in the hippocampus stimulated our exploration of the long-term impact of global ischemia on the GABA pathways within the striatum-substantia nigra system. 

GABA is the major inhibitory neurotransmitter in mammalian brain; disturbance of the GABAergic systems has been linked to both Parkinsonism [11] and epilepsy [12]. The anatomy and function of substantia nigra (SN) has been the focus of decades of research and thousands of publications. Briefly, the substantia nigra (SN) in midbrain is a component of the basal ganglia GABAergic system receiving strong input from the ipsilateral striatum and globus pallidus and plays a pivotal role in the control of movement coordination through inhibition [13]. Neurons within the substantia nigra pars reticulata (SNR) are predominately GABAergic, providing both local inhibitory synapses in the SNR and the adjacent substantia nigra pars compacta (SNC), and powerful inhibition to the thalamus and superior colliculus. In addition to internal segment of the globus pallidus, the SNR is a major output nucleus of the basal ganglia. Inhibitory input to the SNR is derived from the medium spiny neurons of the striatum. Dopaminergic (DAergic) neurons in the SNC release dopamine to synaptic targets including the striatum [13]. 

Animal studies have demonstrated that forebrain ischemia causes postischemic encephalopathy [14] and damage to the GABAergic system [15, 16]. The striatum is vulnerable to transient forebrain ischemia. The dorsolateral striatum has profound neuronal necrosis associated with a marked decrease in GABA synthesis after global ischemia [14, 17]. Extracellular levels of glutamate and GABA increase rapidly following the onset of ischemia and return to baseline quickly [15, 16]. But, multi-ischemic insults instead of a single ischemic event induced a significantly higher extracellular glutamate level and reduced synthesis of GABA, resulting in a significantly higher excitotoxic index (i.e., (glutamate × glycine)/GABA) in the injured regions at 3 hours after reperfusion [15]. The result suggests that decreased synthesis of GABA might contribute to the enhanced ischemic injury in the striatum.

The synthesis of GABA in vertebrates including humans is regulated through the activity of L-glutamate decarboxylases 65 and 67 (GAD65 and GAD67) [18]. GAD is in close association with small vesicles in the axons terminals of neurons. GAD catalyzes the synthesis of GABA from glutamate [18]. GABAergic synaptic interactions with DAergic neurons are present in the substantia nigra pars compacta (SNC) and pars reticulata (SNR). GAD-containing terminals make synaptic contacts with DAergic neurons in SNC and SNR [13]. However, delayed postischemia changes of the GABAergic system within the SN after the moderate single forebrain ischemia have not yet been reported. The presence of Parkinsonism following acute forebrain ischemia prompted us to investigate the long-term changes in the GABAergic system after an ischemic episode. 

We hypothesized that at least part of the cerebral impairment obtained in our previous studies [14, 19] could be related to the damage of the GABAergic system in brain regions critical for motor function, including striatum, primary motor cortex, and substantia nigra. In order to explore this hypothesis, in this study we examined acute and chronic damage to the GABAergic system and coordination of movement after global brain ischemia in adult rats. We analyzed the differences induced by global ischemia with immunohistochemical (IHC) methods with several markers to expand our view of GABAergic system changes after brain ischemia.

## 2. Materials and Methods

### 2.1. Transient Global Forebrain Ischemia

Adult male Wistar rats, weighing 256–405 g and aging 2.8 ± 0.6 months, were subjected to 12.5 minutes (min) of global forebrain ischemia produced by bilateral common carotid artery occlusion plus systemic hypotension (40–50 mm Hg) following an overnight fast. The University of Miami's Animal Care and Use Committee approved all procedures. Anesthesia was induced with 3% halothane with the balance at 70% nitrous oxide/30% oxygen. Animals were intubated endotracheally and ventilated mechanically on a mixture of 0.5% halothane, 70% nitrous oxide, and a balance of oxygen. The femoral arteries were catheterized to permit blood pressure monitoring and arterial sampling for blood-gas measurements. Arterial *P*CO_2_ and *P*O_2_ were maintained in the normal range by ventilatory adjustments. Rats were immobilized by pancuronium bromide, 0.75 mg/kg i.v. Rectal temperature was continually measured and maintained at 37–37.5°C and cranial temperature was separately monitored by a 29-gauge thermocouple implanted into the left temporalis muscle and maintained at 36–36.5°C by warming lamps above the rat's body through the experiment. Blood was withdrawn into a heparinized syringe to reduce the systemic blood pressure to 40–50 mm Hg. Carotid ligatures were tightened for 12.5 minutes, then the ligatures were removed, and the warmed shed blood was reinfused to restore blood pressure to normal. Physiological monitoring was continued for 3 hours into the postischemic period. After the experiment was finished, all wounds were infiltrated with 1% lidocaine. Rats were placed in cages at room temperature with free access to water and food. Sham rats (*n* = 5) received similar surgery but not ischemia [14].

### 2.2. Histopathological and Immunohistochemical Studies

Rats were reanesthetized with halothane and perfused via the ascending aorta with a 2-minute perfusion with physiological saline then FAM (40% formaldehyde, glacial acetic acid, and methanol, 1 : 1 : 8 by volume; final formaldehyde concentration of 4%) for 18 minutes at a pressure of 100–120 mmHg on day 3 (*n* = 5), day 7 (*n* = 7), day 28 (*n* = 6), and day 56 (*n* = 7) after ischemia. The heads were immersed in FAM for an overnight at 4°C. The brains were removed and placed in fresh FAM for an additional day. Coronal brain blocks were embedded in paraffin, and sections (10 *μ*m thick) were prepared at 250 *μ*m intervals. Sections from 3 levels (~0.2, 3.3, and 5.3 mm posterior to bregma) were employed for histological examination [14, 15].

Adjacent sections were stained by hematoxylin and eosin (H&E) or reacted for immunohistochemical visualization of the GABA system. GAD67 is considered as the specific marker for GABAergic neurons and their processes [20]. Mouse anti-GAD67 (MAB5406, Millipore/Chemicon, Temecula, CA) at a working dilution of 1 : 750 [21], antivesicular GABA transporter (VGAT) produced in rabbit (V5764, SIGMA) at 1 : 1500 [22], and rabbit anticalretinin (anti-CR) [SP 13] (ab16694 Abcam) at 1 : 200 were used to detect GABAergic neurons in brain [23]. Antineuronal nuclear antibody (NeuN, MAB377, Chemicon, Temecula, CA) is a specific antibody against neuronal nuclear protein and stains normal neurons in brain at a working dilution of 1 : 200 [24]. Glial fibrillary acidic protein (GFAP, Dako Corp., Carpenteria, CA) was employed to detect active astrocytes at dilution 1: 1500 [14].

Sections were incubated in 1.2% H_2_O_2_ for 20 minutes to block endogenous peroxidase activity, were boiled in 10 mM citrate buffer, pH = 6.0 in a microwave for 15 minutes to retrieve antigen, except those for GFAP labeling [21–24], and, then, were incubated in primary antibodies at room temperature: GAD, CR, and NeuN for 18 hours; VGAT for 1.5 hours; GFAP for 30 minutes. Sections were incubated in the appropriate biotinylated secondary antibodies for 30 minutes. The Vectastain ABC method (Vector Labs, Burlingame, CA) and 3, 3′-diaminobenzidine (DAB) were used for visualization of primary antibody binding. Specific staining was identified by the dark-brown appearance. Two sections were on each slide. The second section was counterstained by hematoxylin to facilitate identification of brain regions.

### 2.3. Immunocytochemistry Controls

For standard immunoperoxidase procedures, two important control slides are included: one for negative control and one for positive control. 

Sections may nonspecifically absorb immunologic reagents to cause false-positive results. For all primary antibodies used, negative controls are accomplished by replacing the primary antibody with sera not containing the primary antibody. To detect the false-positive results, negative controls were conducted with mouse IgG1 (X0931, Dako, Carpenteria, CA) or Rabbit Immunoglobulin Fraction (normal) (X0903, Dako, Carpenteria, CA) to replace the specific primary antibodies. Its negativity validates the positive immunolabeling under the study. Neurons were counted if they exhibited a dark-brown reaction product and had a visible nucleus.

Positive control is for confirming negative reaction to be considered truly negative, not resulting from the reduced sensitivity of the reaction due to improper technique. Positive control is a known histologic slide containing the specific antigen. Preliminary experiments performed with the specific antibodies and NeuN, not related to GABA, were applied to normal (sham) rats. One slide containing NeuN was added. Both GAD or VGAT and NeuN labeling were extensive and intensive, which demonstrated that the right method and skill were employed. Therefore, the subsequent immunohistochemistry studies used only the specific antibody. Slides from sham rats were processed in parallel with sections from postischemic rats as the positive control for the GAD67 and VGAT immunostaining. While sham (positive-control) slide exhibited positive staining, the decreased GAD67 or VGAT immunoreaction implies decreased GABAergic markers in brains from postischemic rats.

### 2.4. Neuronal Counting

We counted the total number of neurons (normal, apoptotic, NeuN positive, or CR positive, as defined below for each study) in each brain region on each hemisphere of a single 10 um coronal brain section; the average of both hemispheres counted as a single data point. Neurons containing a round or ovoid clear nucleus were considered normal (or vital). The degree of ischemic injury was quantified by counting numbers of normal neurons within representative high-power microscopic fields in sections stained with hematoxylin and eosin (H&E). For the striatum (0.2 mm posterior to bregma) and hippocampus (3.3 mm posterior to bregma), we also counted apoptotic neurons as identified by their shrunken and eosinophilic appearance in H&E-stained sections. For the neocortex, we counted all the apoptotic neurons in the full extent of neocortex in three sections 0.2 mm, 3.3, and 5.3 mm posterior to bregma. Apoptotic neurons in the thalamus and hypothalamus were counted in a single section 3.3 mm posterior to bregma. Within the substantia nigra in a single section at 5.3 mm posterior to bregma, we counted apoptotic neurons in H&E-stained sections, and NeuN- or CR-immunopositive neurons [14, 15, 25].

Since the strong immunoreactivity for GAD67 and VGAT is present in the neuronal processes, not somata, it is difficult to count the number of GAD-positive neurons in substantia nigra. Optical density was measured by digitizing the slides using a slide scanner (Pathscan Enabler IV Meyer Instruments) and identical scan settings for all slides. After conversion to 8-bit grayscale images without rescaling, the optical density was calibrated using built-in functions of the image analysis software ImageJ (NIH). To quantify optical density, regions of interest consisting of 0.063 mm^2^ circles were positioned within the SNR. In addition, the loss of GAD67- and VGAT- immunoreactivity in SN was presented by showing the raw images [26].

## 3. Behavioral Testing

### 3.1. Beam-Walking Test

A beam-walking test was applied to assess coordination and integration of motor movements in order to evaluate neurological functional recovery in the rats after forebrain ischemia.

The employed beam-walking apparatus is the typical design for rat to test motor coordination after brain trauma and ischemia [27, 28], which consisted of a plastic beam (1.75 cm wide and 100 cm long, positioned at a height of 65 cm by 2 supporting legs) connected to a platform (12.5 × 12.5 cm) in each end. A black box (W25 × D25 × H20.5 cm) open toward the beam is installed on one platform. Performance was rated on a 0–6 point scale: 0 points if the rat was unable to stay on the beam; 1 point, able only to stay on the beam; 2 points, tried to traverse the beam but fell; 3 points, traversed the beam with more than 50% hind-limb foot-slips; 4 points, traversed the beam with fewer than 50% foot slips; 5 points, only one slip; 6 points, traversed the beam with no slips [27, 28]. We modified the test: when the rat was able to traverse without slipping, that is, reached point 6, the traversal time was recorded by a stopwatch. The time recording began when rat extended a forepaw from the platform onto the beam to start the traverse and stopped when a forepaw was crossing the beam forward into the black box.

### 3.2. Postural Reflex Test

Because macro infarction may appear in striatum and cortex around 1 month or later after the forebrain ischemia [14], a standardized neurobehavioral battery, *the postural reflex test*, applied routinely to test paralysis in rats subjected brain infarction in middle cerebral artery territory was employed in this study on the impact of global ischemia [28]. The rats are held by the tail 50 cm above a table. Intact rats extend both forelimbs toward the table. The rats displaying this behavior are assigned a score of 0. If the rat flexes one forelimb, score 1, then, the rat is given the lateral push test: placing the rat on the table and applying lateral pressure behind the shoulders in the left and right directions. If the rat is unable to resist the force equally in both directions, it receives score 2 [28].

The tests were conducted on days 1, 2, 7, 14, 28, and 56 following ischemia.

## 4. Statistical Analysis

Results were presented as mean ± SD. Data were analyzed by a *t*-test or one-way analysis of variance (ANOVA) followed by Dunnett's test for multiple comparisons, unless otherwise noted. Significance was accepted for *p* < .05.

## 5. Results

### 5.1. Physiological Variables

Preischemic physiological variables were in normal ranges in all groups, and no significant intergroup differences existed (Table 1). 

### 5.2. Neuronal Histopathology

**Figure 1:**
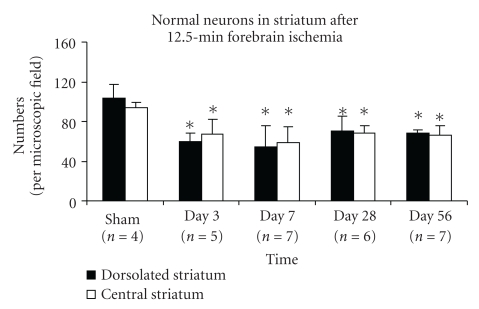
The number of normal (i.e., vital) neurons in the striatum decreases rapidly after global ischemia. Bar graph shows normal neurons (mean ± SD) significantly decreased in rat striatum at days 3–56 after 12.5 minutes of transient forebrain ischemia, per high-power (40 ×) microscopic field (*indicates the significant difference, ischemic versus sham groups, *p* ≤ .03, ANOVA, post hoc Tukey HSD).

**Figure 2:**
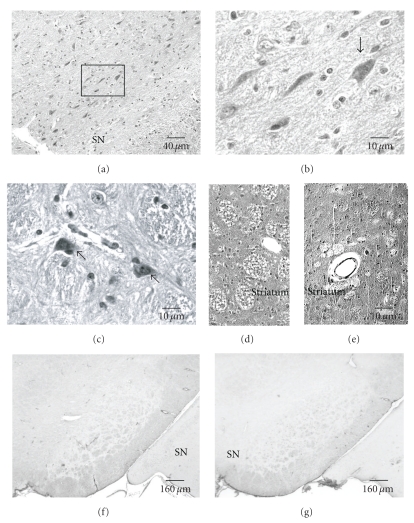
Neurodegeneration in substantia nigra (SN) and striatum at day 56 following 12.5 minutes of transient global ischemia in rats (H&E staining). Eosinophilic and shrunken neurons (arrows) are present in SN ((a)–(c)). (b) is a magnified view of the boxed area in (a). Eosinophilic and shrunken neurons are adjacent to injured blood vessels (c). Striata show severe or moderate spongy changes ((d), (e)) which are proximate to abnormal perivascular area (e). The active astrocytes were detected by anti-GFAP in SN of sham rat (f) and in ischemia rat at day 56 (g). No astrogliosis was observed in both groups.

All rats showed mild necrosis in the dorsolateral striatum, and several rats had mild damage in the central striatum at days 3–7. No additional necrotic injury within the striatum was observed at days 28 and 56. Normal neurons significantly decreased in both dorsolateral and central striatum at days 3–56. The numbers were 60 ± 8 at day 3 (*n* = 5), 54 ± 12 at day 7 (*n* = 7), 70 ± 15 at day 28 (*n* = 6), 68 ± 4 at day 56 (*n* = 7) in dorsolateral striatum and 67 ± 15, 59 ± 16, 69 ± 8, and 66 ± 10 in central striatum while the numbers of normal neurons in sham rats (*n* = 4) were 104 ± 13 in dorsolateral striatum and 94 ± 5 in central striatum. The ischemia reduced the number of normal neurons in striatum (see Figure 1, mean ± S.D., ANOVA, *p* ≤ .01). Moderate-to-severe “spongy” changes (defined as empty spaces in histological sections) in striatum were present at day 56 in 5 of 7 rats (Figure 2(d)). 

The number of normal neurons in hippocampal CA1 sector was significantly reduced from day 3 to day 56. Normal neurons in CA1 are 24 ± 18 (day 3, *n* = 5), 15 ± 8 (day 7, *n* = 7), 24 ± 20 (4 week, *n* = 6) and 21 ± 5 (8 week, *n* = 7); 438 ± 73 (sham, *n* = 4) (*p* < .001, 4 ischemic groups versus sham group). Micro-infarction was seen in CA1 sector at weeks 4-8. 

The cerebral neocortex showed only very mild damage (few or no necrotic neurons) at days 3 and 7, and no necrosis at days 28 and 56. 

No necrotic neurons or infarction was observed in midbrain including the SN at days 3–56. Consistent with this, the total number of NeuN-positive neurons in the dorsal portion of the SN, including SNC and SNL, was 212 ± 42 versus 270 ± 42 (ischemic rats, *n* = 6 versus sham rats, *n* = 3; *p* > .05) at day 56. 

Many eosinophilic and shrunken neurons (indicating apoptosis) were seen in midbrain including substantia nigra (SN) at day 56 (Figures 2(a)–2(c)). Six of 7 ischemic rats displayed apoptotic neurons in SN. Some apoptotic neurons were noticed in the perivascular regions (Figure 2(c)). The numbers of apoptotic neurons in the SN at day 56 are 20 ± 13 (ischemia, *n* = 7) versus 4 ± 3 (sham, *n* = 4, *p* < .05, ANOVA). In the midbrain beyond the SN, 81 ± 70 versus 16 ± 10 were detected (*p* = .05, *t*-test, sham versus ischemia at day 56). These data are consistent with delayed neurodegeneration. Eosinophilic and shrunken neurons were also detected in hypothalamus, thalamus, and cortex at day 56. The numbers (ischemic versus sham) were 54 ± 36 versus 13 ± 11 in hypothalamus (*p* < .05, *t*-test); 113 ± 89 versus 36 ± 10 in thalamus and subthalamus; and 213 ± 140 versus 90 ± 44 in cortex (sum of the cells counted in the 3 standard levels) (*p* > .05, *t*-test, sham versus ischemia at day 56). GFAP-immunoreactivity confirmed that no significant astrogliosis occurred within the SN at days 3–56 (Figures 2(f) and 2(g)). However, the neurons with shrunken cell bodies and condensed-chromatin nuclei were clearly labeled by the antibody against NeuN.

### 5.3. Immunohistochemical Analysis of GABAergic System in Substantia Nigra

**Figure 3:**
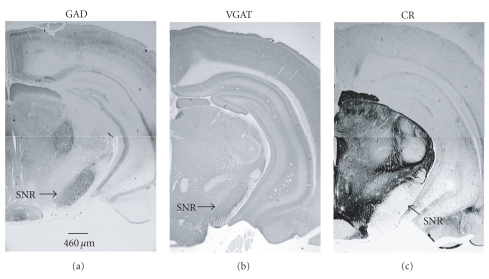
Illustration of GAD67, VGAT, and CR immunostaining in substantia nigra (SN) and the surrounding regions in sham rat on coronal midbrain sections. Immunoreactivity (ir) of GAD and VGAT is high in substantia nigra par reticulata (SNR). The strong CR-ir displays in pars compacta (SNC) and lateralis (SNL) while weak CR-ir present in SNR.

In sham animals, immunoreactivity (ir) for GAD67 and VGAT was dense in the SNR, and CR-ir was highly concentrated in midbrain, especially in the SNC (Figure 3).

### 5.4. Glutamate Decarboxylase 67 (GAD67) Immunocytochemistry

**Figure 4:**
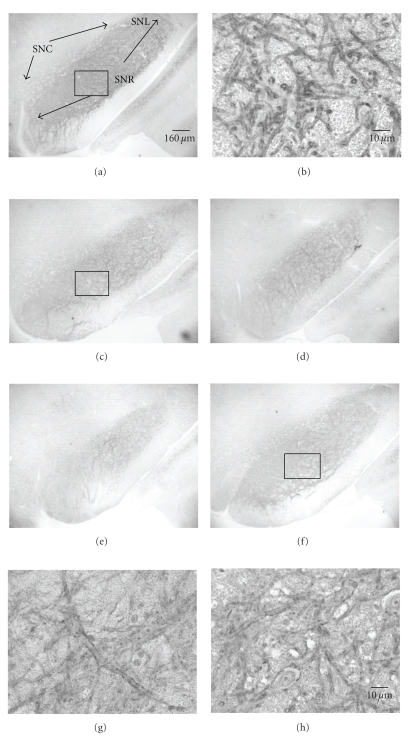
Coronal sections of the ventral mesencephalon showing GAD67-immunoreactivity (GAD-ir) in substantia nigra (SN) at day 56 after 12.5 minutes of global ischemia. The dense GAD-ir is in SN. (a) GAD-ir fiber network is especially prominent in substantia nigra par reticulata (SNR), pars compata (SNC), and pars lateralis (SNL) in sham rats. (b) higher magnification of boxed area in (a). The processes and the cell body are encircled by GAD67^+^ puncta. GAD-ir puncta are closely apposed to GAD-ir dendrites and outline a cell body. Note the GAD67^+^ puncta in contact with the soma and proximal dendrites leaving the cell body. (c)–(f) distribution of GAD-ir from 4 different ischemia rats. (g)-(h) Higher magnification of boxed areas in (c) and (f), respectively. As compare, to sham rats, ischemic rats showed an evident reduction of GAD-ir in SN: the number and density of GAD-puncta are decreased. (b), (g), and (h) are counterstained by hematoxylin.

 Intense GAD67 immunoreactivity (GAD67-ir) was present in the substantia nigra (SN) (Figure 4). The dense and long GAD67 fibers formed a network, which was especially concentrated in SNR and to a lesser extent in SNL and SNC in all of the sham (Figures 4(a) and 4(b)) and ischemic rats (Figures 4(c)–4(h)). At higher magnification, labeled profiles typically contained numerous small clear vesicles. Dense punctate patches of immunoreactivity were observed throughout SN. GAD67-ir was visualized mainly in neuronal processes: axon and dendrites and the puncta were closely apposed to the processes. The processes of GAD67-ir neurons were long and formed a fibrous network within the SNR (Figure 4(b)). 

**Figure 5:**
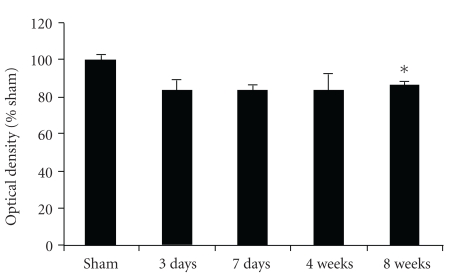
Quantitative analysis of GAD67-immunohistochemistry optical density (mean ± SEM). *indicates the difference from sham rats, *p* < .04, ANOVA, Games-Howell post hoc test).

 Numerous GAD67-ir terminals formed a dense pericellular plexus around immunonegative cell bodies of neurons (Figure 4(a)). Few positively labeled cell bodies were observed. Numerous GAD67 puncta surrounded unstained neurons (Figure 4(b)). The nuclei of neurons remained unstained. Ischemic rats at day 56 displayed decreased GAD67-ir including lesser number of puncta and lower density (Figures 4(c)–4(h)). These qualitative observations supported results using quantification of optical density of the immunostaining (Figure 5). We found a statistically significant decrease relative to sham at day 56 although all the postischemic time points were below the sham values. 

### 5.5. VGAT Immunohistochemical Analysis

**Figure 6:**
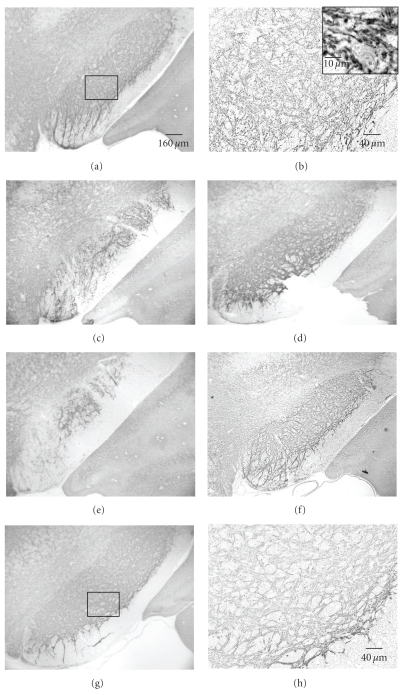
VGAT immunolabeling in SN in coronal midbrain section of rats after 12.5 minutes of global ischemia at day 56. (a) VGAT-ir in sham rat. (b) Higher magnification of boxed area in (a). The insertion with hematoxylin counterstaining presents a higher magnification of VGAT-ir. Immunohistochemical analysis shows abundant puncta of VGAT-positive dots, and some of these puncta encircle an unlabeled neuron's body and its dendrites. (c)–(g): VGAT-ir in 5 ischemic rats at day 56 after injury. (h): higher magnification of boxed area in (g) shows lower density and less amount of VGAT puncta and fiber network than sham rat.

VGAT is reported to selectively identify GABAergic nerve terminals [29]. Immunohistochemical analysis showed numerous VGAT immunopositive puncta in SN from sham animals (Figures 3, 6(a), and 6(b)). Intensive punctuate immunostaining was present in the processes of neurons including axon and dendrites, and the long processes formed a fiber network. Intense VGAT-immunoreactivity (VGAT-ir) was observed in the SNR, SNL, and SNC (Figure 6). VGAT-ir was more intense and uniform in SN in sham rats (Figure 6(a)). At higher magnification, VGAT-ir was primarily detected in small processes but was absent in cell bodies; the puncta encircled neuronal soma (Figure 6(b)). Ischemia appeared to decrease VGAT expression in SN. Ischemic rats showed less puncta and an apparent lower density of VGAT-ir (Figures 6(c)–6(h)). Although VGAT-immunoreactivity appeared significantly less intense in postischemic rats, no significant quantitative differences in gross optical density were observed owing to the high degree of variability in the intensity of immunoreactivity even within the sham group (data not shown).

### 5.6. Calretinin (CR) Immunoreactivity

**Figure 7:**
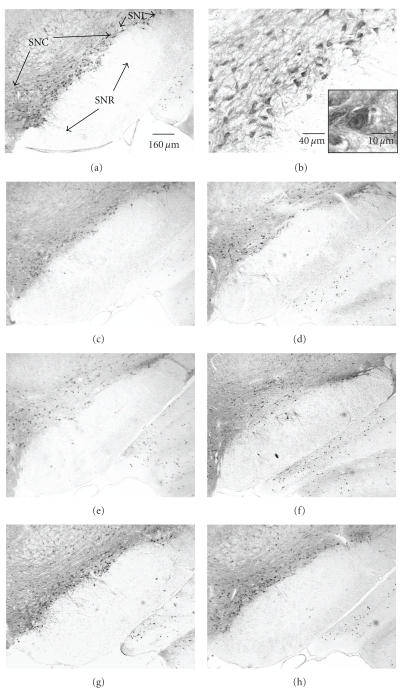
Distribution of calretinin (CR) neurons through substantia nigra (SN) in coronal sections of rats at level ~−5.3 mm to bregma at 56 days of survival. (a) In a sham rat, numerous CR-ir neurons were detected in pars compacta (SNC) and to a lesser extent in pars lateralis (SNL); (b) is the magnification of the boxed region in (a); the insert shows a higher magnification of neurons. (c)–(h) Calretinin immunoreactivity in six ischemic rats after a 12.5-minute period of global ischemia at day 56. The number of CR-neurons was reduced in the SN after ischemia.

**Figure 8:**
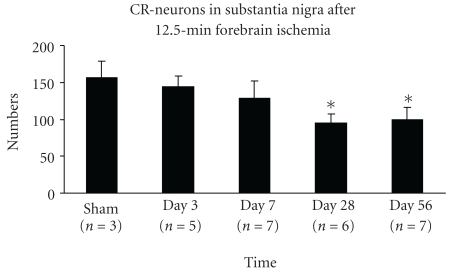
CR-ir neurons are decreased 4–8 weeks after 12.5 minutes of global ischemia (mean ± SD). The bar graph shows the numbers of CR-neurons in the entire SN at level 5.3 mm posterior to bregma are markedly reduced at days 28 and 56 (*indicates the significant difference compared to sham, day 3 and day 7; *p* < .01, ANOVA, post hoc Tukey HSD). No other significant differences were detected.

Calretinin (CR) has been used to detect a subpopulation of GABAergic interneurons [30]. In our study, calretinin-like immunoreactivity was detected in the midbrain at all time points after ischemia. Within the SN, calretinin was exclusively observed in cell bodies and short dendritic processes (Figure 7). In normal rats, CR-immunopositive neurons were densely distributed in SNC, especially in the ventral tier, and many CR-neurons were seen in SN pars lateralis (SNL), which formed a diagonal band-like zone (Figure 7(a)). Several CR-neurons were observed in SNR. During days 3–56 following ischemia, the numbers of CR-neurons progressively declined. The numbers of CR-positive neurons in the entire SNC were 156 ± 23 in sham group (*n* = 3); 144 ± 15 at day 3 (*n* = 5); 128 ± 24 at day 7 (*n* = 6); 95 ± 12 at day 28 (*n* = 6), and 100 ± 16 at day 56 (*n* = 7). Decrease of CR-ir neurons was detected from day 28 through day 56: a 27% decrease from day 3 to day 56 (ANOVA, *p* < .01), a 26% decrease from day 7 to day 28 (*p* = .01), and a 18% decrease from day 7 to day 56 (*p* < .05) was observed. No significant differences were detected between the day 3 and day 7 or between days 3–7 and sham (*p* > .05) (see Figure 8). These data demonstrate a delayed loss of CR-ir beginning after day 7.

### 5.7. Behavioral Analysis

 All of the 30 rats participated in the study received the following behavioral tests.

#### 5.7.1. Beam-Walking Test (BWT)

Rats were trained to walk on the beam to assess the rats ability to maintain balance before surgery. First, rats were put on the platform. All of rats moved around slowly on the platform for 10–15 minutes and, then, began to try to traverse. They walked on the beam faster and faster during the training. Finally, rats ran easily along the beam without faults. They appeared to well tolerate the beam-walking test because they voluntarily repeated the traversing, making round-trips between the black box and the platform during the training. The last tests were done on day 56 following ischemia (*n* = 7). Sham rats (*n* = 5) were also tested. 

**Figure 9:**
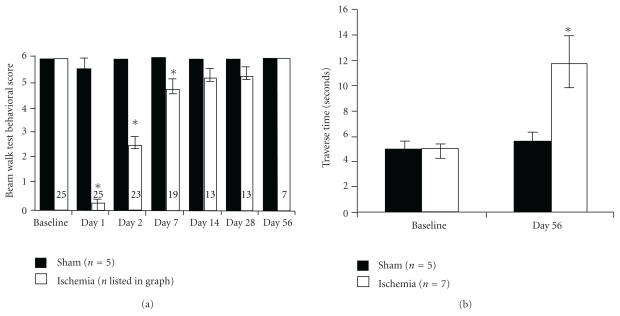
Functional recovery after global ischemia was tested using the beam-walking test (BWT). (a) The mean neurological scores (a score of 6 equals normal behavior) by beam-walking test before and after ischemia at days 1, 2, 7, 14, 28, and 56. The ability of the rats to walk on the beam is decreased during the acute phase after global ischemia but gradually recovers (mean ± SEM; *p < .01, paired two-tailed *T*-Tests assuming unequal variances). (b) The time for beam traverse before and at day 56 after ischemia. Despite the improvement in the qualitative scoring (i.e., no foot faults), the ischemic rats were much slower than sham or baseline. Because the ischemic rats were not able to complete the traverse until day 56, these are the only time points available. *indicates significant difference between preischemia and postischemia in (a) and the difference between sham and ischemia group at day 56 in (b) (mean ± SEM; *p* < .02; ischemia day 56 versus either sham or baseline, ANOVA, Tukey HSD post hoc test between all four groups).

 After training, the preischemia traversing beam score was 6 for all 30 rats. The traversing score of sham rats reached 6 point (no faults) at 48 hours after surgery while ischemic rats did not reach 6 until day 56. Compared to sham rats, the ischemia rats had significantly lower scores at days 1, 2, and 7 after the insult (see Figure 9(a)). At day 56, the score of ischemia group reached 6; however, the ischemic rats traversed slower than sham rats, and the mean time for traverse was 11.8 ± 5.4 (ischemia, *n* = 7) versus 5.6 ± 1.2 seconds (sham, *n* = 5) (Figure 9(b)). Compared to preischemia the rats walked significantly slower at day 56: the traverse time was 4.88 ± 0.92 versus 11.8 ± 5.4 seconds (postischemic group, baseline (day 0 versus day 56; Figure 9(b)). However, sham rats did not traverse slower in day 56 than presurgery (5.16 ± 0.66 versus 5.6 ± 1.2 seconds; sham group, baseline versus day 56). This indicates ischemia rats took more time to finish traverse due to brain injury, not aging or surgery.

#### 5.7.2. Postural Reflex Test

The test was conducted on days 1, 2, 7, 14, 28, and 56 before BWT. All of rats scored 0 (no deficit) during the whole 8 weeks after ischemia. This is consistent with our histological analysis and demonstrates the lack of macroinfarction.

## 6. Discussion

This study offers evidence that the GABAergic system in substantia nigra (SN) underwent delayed degeneration at 4–8 weeks following a single episode of 12.5-min forebrain ischemia, including a decrease of GAD67-, VGAT- and CR- immunoreactivity; no direct ischemic injury was detected in the SN during the acute postischemic stage (postinjury days 3 and 7). The behavioral recovery was assessed with the beam-walking test to detect motor coordination revealing a delayed and attenuated recovery. Thus, the present study extended our understanding of delayed postischemic encephalopathy. 

We demonstrated that the substantia nigra contains a high density of immunoreactivity for markers of the GABAergic system in normal rats. This is similar to previous observations [13, 26]. A previous study reported that neurons in SN are resistant to a single moderate episode of ischemia. SN damage was not observed at the first 7 days [31]. A severe (20-minute) forebrain ischemia may induce delayed regression in SNR at 3 weeks, which depended on the extent of initial striatal damage [32]. This shows that moderate ischemia induced SN degeneration at the 4th week while mild-to-moderate injury was detected in striatum at days 3–7 after the insult. Evidence for substantia nigra regression included reduction in the density of GAD67 and VGAT fiber networks in SNR and a decline in numbers of CR-neurons in SNC. This degeneration might be the regression secondary to the preceding striatal injury and might be induced by injured blood vessels since degeneration appeared in some cases adjacent to abnormal perivascular regions [19], and endothelial degeneration persists in brain for at least 10 weeks [14]. 

### 6.1. Ischemia Reduces GAD67 Expression in SN

GAD67-ir profiles observed in sham animals in the present study are similar to previous studies [13, 33–36]. GAD67 immunocytochemistry is used to be studied by labeling the GABAergic neurons and synapses [26, 37] and has been extensively used to identify GABAergic populations throughout the brain [38]. Greater concentration of GAD67-ir has been previously associated with the increased GABA synthesis [18]. Electron microscopic and light microscopic studies demonstrated that GAD67 puncta are indeed in axon terminals, synaptic vesicles, and dendrites but not in neuronal soma in substantia nigra and other regions [13, 34–36]. 

GABAergic efferent neurons in striatum are highly vulnerable to ischemia; GAD67-immunoreactivity markedly decreases associated with the death of striatal efferent neurons in the lesioned dorsal striatum and markedly decreases the GABA synthesis in SRN induced by global ischemia [17]. In addition, striatal ischemia induced by middle cerebral artery occlusion (MCAO) leads to a robust decrease of GAD67 immunoreactivity in the ipsilateral SNR for at least 94 days [26]. 

Measurement of cellular GAD expression is crucial for the present study. Because of the difficulty of acquiring protein samples from small, specific brain regions for Western blots, previous studies have relied on in situ hybridization (ISH) for isoforms of GAD mRNA or GAD protein immunohistochemistry (IHC) as markers for cell counts. These studies found that the number of neurons expressing GAD67 mRNA is significantly less than the number of neurons expressing GAD67 protein [21], suggesting that for this protein immunohistochemistry may be more reliable than mRNA detection. Thus, IHC was chosen in the present study. Based on GAD67 immunoreactivity, GAD expression in SN was decreased at the 8th week after 12.5 minutes of transient forebrain ischemia.

### 6.2. Ischemia and VGAT Expression in SN

Vesicular GABA transporter (VGAT) is an integral membrane protein and a neurotransmitter transporter in GABAergic neurons [39]. Recent studies reported that focal brain ischemia decreases VGAT in the injured brain regions during the 72 hours after reperfusion [40]. VGAT mediates the transport of GABA from cytosol into synaptic vesicles [40]. VGAT protein-containing synaptic terminals, neuropil, and somata were visualized by IHC. The VGAT-ir in our study is similar to the VGAT labeling observed in a previous study [41]. GAD 67 and VGAT couple in synaptic vesicles (SVs). GABA synthesized by GAD67 is transported into SVs by VGAT [41]. Inhibiting GAD decreases VGAT activity [41]. This may explain why the two different GABA cytological markers, GAD67 and VGAT, showed similar topographic distributions in this study. VGAT-ir in the entire SNR appeared clearly less intense in ischemic rats in the present study, but the high degree of variability in gross optical density in the regions of interest (ROI) of sham group resulted in no significant differences on VGAT-ir. Neurotransmitters released into the synaptic cleft will be cleared by active reuptake into the surrounding neurons, which is mediated by plasma membrane transporters [42]; and the transport from cytosol into synaptic vesicles is mediated by vesicular transporters in synaptic vesicles [40]. Decrease of nigral GAD-expression should result in the reduction of GABA synthesis and release in SN, which may decrease the inhibition on DAergic neurons to play a role in Parkinsonism development after forebrain ischemia.

### 6.3. Ischemia Reduces Calretinin (CR) Immunoreactive GABAergic Neurons in SN

The present study found that the CR-neurons in SN decreased at weeks 4 and 8 after 12.5 minutes of forebrain ischemia. However, the anti-NeuN immunolabeling failed to detect the neuronal reduction within the SN, owing to the numerous cell types within this region and the relatively small number of CR-neurons. Another possibility is that apoptotic and necrotic neurons can be labeled by the NeuN antibody (neuron-specific nuclear antigen). In the present study, we observed that some neurons with apoptotic features (Figures 2(a)–2(c)) were labeled by the NeuN antibody. This suggests that NeuN-positive staining of injured neurons may occur if the nuclei have not been phagocytosed or otherwise removed. We believe that the apoptotic neurons were stained by NeuN antibody but were not labeled by CR antibody, likely due to a rapid loss of CR protein after injury. Thus, the loss of CR-ir neurons may be reflected in the apoptotic neurons detected by NeuN.

GABA neurons can be divided into different subgroup based on their expression of calcium-binding proteins: calretinin (CR), calbindin (CB), and parvalbumin (PV) [30]. Although calcium binding proteins are hypothesized to maintain calcium-homeostasis, a recent study suggests that calcium-binding proteins do not confer resistance to ischemic injury [17]. Damage to striatonigral pathway leads to the subsequent striatal decline of GABA release and progressive GABAergic neuronal loss, which contributes to a delayed and progressive loss of neurons in SNR by a decrease in the inhibitory striatal input [43]. In SNC, about 50% of CR-positive neurons are GABAergic neurons and 50% are others [44, 45]. 

Dopaminergic (DAergic) neurons do not appear to be selectively vulnerable to ischemia [26]. In rats, striatal ischemia induced by 2-hour MCAO kills striatal neurons and leads to a tremendous decrease of GABAergic pathway in the ipsilateral SNR at days 3–94 following ischemia while not reducing DAergic neurons in SNC [26]. Since the striatal injury induced by 12.5-minute forebrain ischemia is much milder than than by the 2-hours MCAo, logically the forebrain ischemia in the present study should not decrease the DAergic neurons in SN. Calretinin provides protection against degeneration of DAergic neurons in SNC [25, 45]. In Parkinson's disease (PD) patients, the CR-positive DAergic neurons appear to be less vulnerable to neurodegeneration [25]. In the postischemic/hypoxic parkinsonism patients, SNC is spared from the injury (see Section 6.7 below). Thus, we assume the diminished CR-ir neurons in SNC in the present study to be GABAergic.

### 6.4. GABA System Controls Activity of DAergic Neurons

Parkinson's disease (PD) is associated with the progressive loss of dopaminergic neurons in substantia nigra pars compacta (SNC) and their axons projecting to striatum by unknown causes [46]. However, DAergic neurons are under GABAergic system control. A strong GABAergic input via striatonigral pathway innervates the DAergic neurons and is central to normal function of this complex neuronal system [13]. 

DAergic neurons are concentrated in SNC and involved in the feedback loops within the basal ganglia [47]. The GABAergic pathway exerts a direct regulatory effect on the activity of nigral DAergic projection [48]. The striatal dopamine release is modulated by ipsilateral intranigral GABA via direct action on the nigraostriatal projection [48]. Thus, decrease of GABA in the SN will influence the amount of dopamine in the striatum in the present study.

 GABAergic inputs within the SN originate from striatum, globus pallidus, and local nigral neurons [26]. The majority of the striatonigral pathway is the afferent projection into the SNR from the GABAergic efferent neurons in striatum. The local nigral GABAergic neurons in the SNR and SNC strongly inhibit DAergic neurons in the SNC [49]. Recurrent inhibition is a hallmark of DAergic firing patterns and dopamine release in the striatum [49]. In addition, globus pallidus (GP) exerts powerful control over the SNR input and subsequent control over the DA neurons via SNR [49]. Decrease of GABAergic afferent input to the SNR results in the disinhibition of GABAergic neurons in SNR and a subsequent decrease in the firing DAergic neurons whereas increase of burst firing in DAergic neurons is associated with a significant increase of extracellular dopamine in striatum [49]. Thus, the degeneration of GABA system in SN might decrease the dopamine (DA) in striatum through the disinhibition of SNR GABAergic neurons to reduce the striatal DA and subsequently induce the inhibition of movement. In future studies, the amount of DA and GABA in striatum and SN after ischemia should be assessed. Function studies using electrophysiological approaches will be required to fully elucidate the impact of delayed GABAergic degeneration after global ischemia.

The DAergic neurons in the SNC and SNR receive dense synaptic input from GABAergic terminals [47]. More than 70% of the terminals afferent to the DAergic neurons in SNC are GABAergic the major synaptic input is derived from striatum via striatonigral pathway; striatonigral terminals form the synapses at the dendrites and perikarya of DA neurons in the whole substantia nigra [47]. Approximately 80% of the GABAergic synapses are on the perikarya and large dendrites [13]. Therefore, DAergic neurons are under a powerful inhibitory control from the GABAergic system. After global ischemia, a decrease of the striatonigral efferent to SN may influence the function of DAergic neurons, which in turn might lead to the disturbance of dopamine release in striatum and interfere with motor coordination [49].

### 6.5. Functional Considerations

**Degeneration of GABA System May Influence the Function of DAergic Neurons**

The basal ganglia, of which the striatum (caudate nucleus and putamen) and SN form a functional unit, are a motor coordination circuit. Degeneration of the DAergic projection from the SNC to the striatum is directly involved in the development of classic parkinsonian motor symptoms. The substantia nigra reticulate (SNR) is one of two major output nuclei of the basal ganglia. Normal motor activity is dependent on coordinated striatonigral and striatopallidal output, which regulated substantia nigra output, which in turn regulates dopaminergic substantia nigra output [50]. The output neurons of striatum use GABA as their principal transmitter colocalized with the neuropeptides enkephalin (*Enk*) or substance P (*SP*)/dynorphin (*Dyn*). Striatal neurons projecting directly to the output structures use GABA, *SP*, and *Dyn* while neurons projecting to the globus pallidus (GP) use GABA and *Enk* [51]. Striatal neurons that express dynorphin (*Dyn*) and substance P (*SP*) provide an inhibitory input to SNR. Striatal neurons containing enkephalin (*Enk*) provide an inhibitory input to the GP. Pallidal neurons provide an inhibitory input to subthalamus. The subthalamic provides excitatory input to SNR. GABAergic projection neurons in SNR inhibit the neurons in thalamus which in turn project to posterior regions of the frontal lobe (motor-related), superior colliculus, and pedunculopontine nucleus (PPN) to lead a normal behavioral activity [51, 52]. These nuclei form the basal ganglia-thalamo-cortical network, motor circuit. Neurons expressing *Enk* and *SP* are located in dorsal striatum. All of the *Enk-, Dyn-*, and *SP-*expressing neurons use GABA as a transmitter [50]. Dopamine exerts different effects on pallidal-projecting/*Enk-expressing neurons*, *and nigral-projecting/SP-* and* Dyn-*expressing striatal neurons [50]. The neurons in dorsal striatum are more vulnerable to ischemia than others. GABAergic efferent neurons expressing *Enk* and *SP* are greatly decreased by ischemia [17]. Global brain ischemia significantly injures neurons in dorsal striatum while GP appears unaffected [14, 19]. The clinical appearance of movement disorders correlates with the striatal dopamine reduction owing to the DAergic neuronal loss in Parkinson's disease [50, 51]. But, striatal ischemia does not reduce DAergic neurons. Ischemia reduces the dopamine's targets and decreases the GABAergic inputs in SN [26]. Whether the ischemic injury can initiate a disturbance of striatal dopamine release to induce the motor inhibition needs to be investigated in the future studies. However, the reduction of GABA in SNR may produce motor inhibition, dyskinesias [51, 52]. Therefore, the retrograde degeneration of GABA system in SN might reduce GABA in SNR. Combined with a 30%–40% of normal-neurons loss in dorsal striatum produced by ischemia, impaired motor control may be a direct consequence of the degeneration of GABA systems in the SN. Behavioral impairment could be related to the damage of the other brain regions critical for motor (primary motor cortex). However, there was no paralysis in the experimental rats; thus, the pyramidal system function seemed intact. The animals slowness on beam traverse implies that postischemic animals might be hampered by the disturbance of coordination, that is, bradykinesia, due to significant GABA system degeneration in the SN and neuronal loss in striatum after transient global ischemia. It should be noted that Huntington's disease (HD) is associated with excitotoxic neurodegeneration of medium spiny neurons (MSNs) in the striatum. These neurons are also acutely vulnerable to ischemia. Loss of these MSNs in HD is associated with chorea. Our results also show striatal degeneration in addition to the diminished GAD67 immunoreactivity in the SNR while previous studies suggest no loss of dopamine neurons in the SNC. In this context, we propose that the combination of GABAergic degeneration in the SNR and other targets of global ischemia gives rise to a net loss of dopamine release in the striatum and may represent an alternative path to Parkinson-like symptoms in the absence of overt loss of dopamine neurons.

### 6.6. Striatal Ischemia Produces Nigral Retrograde Degeneration in Humans

In humans, striatal infarction, not cortical infarction, leads to the retrograde degeneration in the ipsilateral substantia nigra, an area remote from the infarct and unlikely to be directly influence by ischemia. This similarly occurs in rat after occlusion of middle cerebral artery. The degeneration appears at day 14 after stroke on average in patients [53, 54]. Since GAD67 [55] and calretinin [23] are found in human brain, the present study can be considered comparable to the pathological process in humans. 

### 6.7. Postischemic/hypoxic Parkinson's Syndrome in Patients

Parkinsonism, a movement disorder, has been observed in the patients awoke from the global brain ischemia or hypoxia induced by cardiac arrest (CA) resuscitation [56–60], heart surgery [6], and hypoxia, including carbon monoxide (CO) poisoning [61–66]. The symptoms, including rigidity, masked face, short-step gait, and akinesia/bradykinesia, show up at 7–40 days after the insults and may spontaneously and completely recover or with only mild sequelae within about 1 year with no dopaminergic medication [59, 61, 63, 64].

Bilateral pallidal necrosis or infarction is the characteristic lesion of the parkinsonism induced by postischemic/hypoxic encephalopathy. The typical lesion in globus pallidus (GP) is well defined and usually extends into internal capsule [56, 57, 59]. Some patients has the entire lentiform nucleus (GP and putamen) injury and massive cerebral white matter damage, comprised of cytotoxic edema and diffuse demyelination/axonal destruction, as well as cortical lesions [61, 64–67]. Magnetic resonance imaging (MRI) [56, 57, 59, 64, 66–69] and autopsy [56, 59, 64, 66, 68, 69] demonstrated that the postischemic/hypoxic parkinsonism almost always have bilateral basal ganglia necrosis/infarction, but substantia nigra lesions are very rare [66]. In a literature review, 2 of 86 patients with the parkinsonism acquired from CA- or CO-intoxication had SN injury: in one patient, MRI and autopsy revealed the bilateral SNR (SNC was spared) and GPs necrosis [70]; in a second patient, MRI disclosed bilateral pallidal and substantia nigral lesion [71]. In addition, MRI revealed that one such patient had only the periventricular and deep white matter change no basal ganglia injury [65]. 

Pallidal injury results in akinetic-rigid syndrome [59]. Delayed demyelination and axonal destruction also contribute to developing the basal ganglia deterioration [59]. Although the white matter impairment is reversible, its amelioration and disappearance is in accordance with the complete recovery of the akinetic-rigid syndrome [64, 65]. Reduction of putaminal dopamine uptake due to the impairment of presynaptic DAergic activity in putamen (as opposed to DAergic neuronal loss) is responsible for the development of postischemic/hypoxic parkinsonism [64, 67]. 

The basal ganglia are especially vulnerable to hypoxia [59]. The regional cerebral blood flow (rCBF) is reduced in the globus pallidus, the frontal and temporal cortex, and the cerebral white matter during the delayed encephalopathy [63]. Hypoxia-ischemia typically produces lesions in the globus pallidus; and vascular insufficiency is a major mechanism [56, 68].

The parkinsonism recovery is associated with the healing or amelioration of injury in white matter and GPs [61, 63, 64, 67]. Consistent with this, levodopa anticholinergic drugs are ineffective [56, 57, 61, 63]. 

Bhatia and Marsden studied 240 patients with motor or behavioral disorders acquired from hypoxia and found that bilateral lesions of the lentiform nuclei, either of the globus pallidus or the putamen, caused parkinsonism without SNC neurodegeneration [72]. Lesions in the globus pallidus external (GPe) and internal (GPi) divisions lead to the akinetic-rigid syndrome in men [73].

In conclusion, we detected deterioration of the GABAergic system in substantia nigra pars reticulata (SNR) after the moderate forebrain ischemia, which began at 4 weeks. This was associated with a slowing of the traversal of the beam-walking test at 8 weeks, despite the observation that all the rats could perform the test without fault. The coexistence of the neuronal loss of bilateral striatum, the delayed degeneration of GABAergic system in SNR and the disturbance of coordination, bradykinesia, provided in the present study therefore may partly explain the Parkinsonism development without DAergic neuronal loss in substantia nigra par compata (SNC) in the postischemic/hypoxic encephalopathy in patients. Thus, neuroprotective strategies aimed at preserving GABAergic function within the substantia nigra and improving rCBF in basal ganglia may alleviate parkinsonism in the delayed encephalopathy.

## Figures and Tables

**Table 1:** Physiological variables in rats with 3–56 days of survival. Values represent mean ± SD (*MAP,* mean arterial blood pressure).

Group	*n*	Weight (g)	Age (month)	MAP (mm Hg)	pH (units)	*p*CO_2_ (mmHg)	*p*O_2_ (mm Hg)	Glucose (mg/dl)
3 day	5	365 ± 10	3.4 ± 0.4	127 ± 12	7.390 ± .04	41.1 ± 3.1	123.8 ± 23.7	160 ± 17
7 day	7	322 ± 49	3.0 ± 0.4	125 ± 11	7.374 ± .04	41.2 ± 3.5	148.2 ± 29.0	145 ± 15
4 week	6	288 ± 29	2.7 ± 0.2	133 ± 12	7.374 ± .04	39.5 ± 4.1	145.0 ± 17.8	144 ± 24
8 week	7	319 ± 46	2.7 ± 0.5	129 ± 10	7.428 ± .08	38.2 ± 2.7	126.1 ± 17.2	139 ± 23
Sham	4	304 ± 23	2.7 ± 0.4	137 ± 11	7.395 ± .05	39.8 ± 4.2	157.0 ± 16.4	142 ± 14
